# Impairment of bile acid metabolism by perfluorooctanoic acid (PFOA) and perfluorooctanesulfonic acid (PFOS) in human HepaRG hepatoma cells

**DOI:** 10.1007/s00204-020-02732-3

**Published:** 2020-04-06

**Authors:** Anne-Cathrin Behr, Anna Kwiatkowski, Marcus Ståhlman, Felix Florian Schmidt, Claudia Luckert, Albert Braeuning, Thorsten Buhrke

**Affiliations:** 1 Department of Food Safety, German Federal Institute for Risk Assessment, Max-Dohrn-Str. 8-10, 10589 Berlin, Germany; 2 Wallenberg Laboratory, Department of Molecular and Clinical Medicine, Sahlgrenska Academy, Gothenburg University, 413 45 Gothenburg, Sweden; 3 Signatope GmbH, 72770 Reutlingen, Germany

**Keywords:** Liver toxicity, Cholesterol, Bile flow, Contaminants, Hepatocytes

## Abstract

Perfluorooctanoic acid (PFOA) and perfluorooctanesulfonic acid (PFOS) are man-made chemicals that are used for the fabrication of many products with water- and dirt-repellent properties. The toxicological potential of both substances is currently under debate. In a recent Scientific Opinion, the European Food Safety Authority (EFSA) has identified increased serum total cholesterol levels in humans as one major critical effect being associated with exposure to PFOA or PFOS. In animal studies, both substances induced a decrease of serum cholesterol levels, and the underlying molecular mechanism(s) for these opposed effects are unclear so far. In the present study, we examined the impact of PFOA and PFOS on cholesterol homoeostasis in the human HepaRG cell line as a model for human hepatocytes. Cholesterol levels in HepaRG cells were not affected by PFOA or PFOS, but both substances strongly decreased synthesis of a number of bile acids. The expression of numerous genes whose products are involved in synthesis, metabolism and transport of cholesterol and bile acids was strongly affected by PFOA and PFOS at concentrations above 10 µM. Notably, both substances led to a strong decrease of CYP7A1, the key enzyme catalyzing the rate-limiting step in the synthesis of bile acids from cholesterol, both at the protein level and at the level of gene expression. Moreover, both substances led to a dilatation of bile canaliculi that are formed by differentiated HepaRG cells in vitro. Similar morphological changes are known to be induced by cholestatic agents in vivo. Thus, the strong impact of PFOA and PFOS on bile acid synthesis and bile canalicular morphology in our in vitro experiments may allow the notion that both substances have a cholestatic potential that is connected to the observed increased serum cholesterol levels in humans in epidemiological studies.

**Electronic supplementary material:**

The online version of this article (10.1007/s00204-020-02732-3) contains supplementary material, which is available to authorized users.

## Introduction

Perfluoroalkylated substances (PFAS) are man-made chemicals that are used for numerous industrial applications, e.g., for the fabrication of surface coatings with water- and dirt-repellent properties. PFAS are characterized by their extraordinary stability; they are resistant against thermal, chemical and biological degradation and, therefore, persist in the environment (Lau et al. 2007). Perfluorooctanoic acid (PFOA) and perfluorooctanesulfonic acid (PFOS), the most prominent members of the PFAS family, are toxicologically well characterized. In rodents, both substances have been shown to be hepatotoxic which is predominantly associated with their capability to activate the peroxisome proliferator-activated receptor alpha (PPARα) (Kennedy et al. 2004; Bosgra et al. 2005). However, this molecular initiating event is presumably more relevant for rodents rather than for humans (Palmer et al. 1998; Takacs and Abbott 2006; Wolf et al. 2008; Vanden Heuvel et al. 2006). Humans are exposed to PFOA and PFOS via contaminated drinking water, food and dust. After oral ingestion, both substances are readily resorbed into the body where they accumulate in the blood by binding to serum proteins. Furthermore, PFOA and PFOS are slowly excreted via urine and feces leading to high serum half-lifes of 2–4 years for PFOA and about 5 years for PFOS (EFSA et al. 2018; Olsen and Zobel 2007).

In 2018, the European Food Safety Authority (EFSA) published a Scientific Opinion on the risk to human health related to the presence of PFOA and PFOS in food (EFSA et al. 2018). In this Scientific Opinion, risk assessment was based on human epidemiological data rather than on the results of animal studies. Based on these human data, a provisional tolerable weekly intake (TWI) of 6 ng/kg body weight per week was established for PFOA, and a TWI of 13 ng/kg body weight per week was derived for PFOS. For both substances, the increase of serum total cholesterol was identified as the most critical effect. In addition to this, a decrease in antibody response after vaccination was identified as a critical endpoint for PFOS and increased serum alanine transaminase levels were considered as a relevant effect mediated by PFOA. The observation of reduced birth weights was also taken into account for both substances.

The epidemiological observation that PFOA and PFOS exposure correlates with increased serum total cholesterol levels in humans is opposed to the results obtained with animal studies as both substances were shown to decrease serum cholesterol levels in rodents (Elcombe et al. 2012; Minata et al. 2010; Wang et al. 2014) which points to species differences regarding the impact of PFOA and PFOS on cholesterol homeostasis. These species differences and the underlying molecular mechanism(s) being responsible for the opposed effect of PFOA and PFOS on serum cholesterol levels in humans compared to rodents are currently under debate.

With a focus on the human system, we have used in the present study the HepaRG cell line as an in vitro model for human hepatocytes to examine the impact of PFOA and PFOS on cholesterol and bile acid metabolism at the cellular and at the molecular level. Our in vitro data indicate that both substances have an impact in particular on bile acid metabolism, allowing the conclusion that high concentrations of PFOA or PFOS may trigger cholestasis in humans in vivo.

## Materials and methods

### Chemicals

Perfluorooctanoic acid (PFOA) and cyclosporine A (CSA) were purchased from Sigma-Aldrich (Taufkirchen, Germany) and perfluorooctanesulfonic acid (PFOS) was obtained from ABCR GmbH (Karlsruhe, Germany) in the highest available purity.

### Cell culture conditions

HepaRG cells were purchased from Biopredic International (St. Grégoire, France) and cell culture was conducted according to the manufacturer’s protocol. Briefly, HepaRG cells were maintained in William’s E medium (PAN Biotech, Aidenbach, Germany) supplemented with 10% (v/v) fetal bovine serum (FBS, PAN Biotech), 5 µg/mL insulin (PAN Biotech), 50 µmol/L hydrocortisone hemisuccinate (Sigma-Aldrich), 100 U/mL penicillin and 100 µg/mL streptomycin (Capricorn Scientific, Ebsdorfergrund, Germany) at 37 °C in a humidified atmosphere containing 5% CO_2_. After seeding of the desired cell density, HepaRG cells were cultivated for 14 days. The initiation of cellular differentiation was carried out by adding 1% (v/v) dimethyl sulfoxide (DMSO) to the medium for 2 days, followed by an addition of 1.7% (v/v) DMSO to the medium for up to day 28. The medium was changed every 2–3 days. Subsequently, the differentiated HepaRG cells were starved for 2 days in serum-free medium consisting of William’s E medium phenol red-free (PAN Biotech), 1% (v/v) insulin–transferrin–selenium (ITS, Capricorn Scientific), 5 µg/mL insulin, 50 µM hydrocortisone hemisuccinate, 0.1 mg/mL fatty acid-free bovine serum albumin (BSA, Sigma-Aldrich), 100 U/mL penicillin and 100 µg/mL streptomycin.

HepG2 cells (European Collection of Cell Cultures, Porton Down, UK) were cultivated in Dulbecco’s modified Eagle’s medium (DMEM; PAN Biotech) supplemented with 10% (v/v) FBS (PAN Biotech), 100 U/mL penicillin and 100 µg/mL streptomycin (Capricorn Scientific, Ebsdorfergrund, Germany) at 37 °C in a humidified atmosphere containing 5% CO_2_.

### Viability assay

Cellular viability was determined using the 3-(4,5-dimethylthiazol-2-yl)-2,5-diphenyltetrazolium bromide (MTT) assay as previously described (Scharmach et al. 2012). HepaRG cells were differentiated in 96-well plates, followed by a treatment with PFOA and PFOS or CSA (20 µM) for 24 h or 48 h. The assay was conducted in quadruplicates with at least three individual experiments. Untreated cells served as control, and medium containing 0.01% Triton-X 100 served as positive control.

### Gene expression analysis

For gene expression analysis, HepaRG cells were differentiated in 6-well plates and treated with the test compounds for 24 h or 48 h. After treatment, cells were washed twice with ice-cold phosphate-buffered saline (PBS), and RNA was extracted using the RNeasy Mini Kit (Qiagen, Hilden, Germany) following the manufacturer’s protocol. Total RNA was quantified with a spectrophotometer (Nano Drop 1000; Nanodrop Technologies, Wilmington, USA). Synthesis of cDNA was conducted using the High Capacity cDNA Reverse Transcription Kit (Applied Biosystems, Foster City, USA). Real-time qPCR was performed on an ABI 7900HT (Thermo Fisher Scientific, Waltham, USA) using Maxima SYBR Green/ROX qPCR Master Mix (Fermentas, St. Leon Rot, Germany). PCR products were verified by melting curve analysis. Relative changes in mRNA transcription levels were quantified using the 2^−ΔΔCt^ method (Pfaffl 2001) normalized to *GAPDH*. Primer sequences are listed in Table S1. Three individual experiments were performed.

### Reporter gene assay

Impact of PFOA and PFOS on *CYP7A1* promoter activity was assessed via a luciferase-based reporter gene assay. Following the principle of sequence and ligation-independent cloning (Li and Elledge 2007; Jeong et al. 2012), the plasmid pGL4.14–CYP7A1-Prom was constructed by cloning the promoter of *CYP7A1* into the vector pGL4.14 upstream of the firefly luciferase reporter gene (Promega, Madison, WI, USA). First, the vector pGL4.14 was linearized by digestion with restriction enzymes *Nhe*I and *Xho*I. The *CYP7A1* promoter region (-2014 to -1 bp from translation start site) was amplified from human genomic DNA isolated from HepG2 cells using the primers 5′-CGG TAC CTG AGC TCG CTA GCC AGG AAA GAA CTG CAC CCA TAA T-3′ (forward) and 5′-CAG ATC TTG ATA TCC TCG AGT TTG CAA ATC TAG GCC AAA ATC T-3′ (reverse) containing at the 5′-end a 20-bp region homologous to the linearized ends of the vector (underlined). Both linearized vector and amplified DNA insert were treated with T4 DNA polymerase to generate homologous 5′-overhangs. Following annealing of the homologous single strands, the annealed DNA complex was directly transformed into competent *E. coli* cells where homologous recombination in vivo leads to the filling of gaps previously generated by excessive exonuclease activity and the joining of annealed strands. HepG2 cells were seeded at a density of 2 × 10^4^ cells/well on 96-well plates. After 24 h, the cells were transiently transfected for 6 h with the *CYP7A1* promoter plasmid pGL4.14–CYP7A1-Prom and the *Renilla*-luciferase construct pcDNA3-Rluc (Luckert et al. 2013) for normalization using the TransIT-LT1 transfection reagent (Mirus Bio, Madison, USA) according to the manufacturer’s protocol. Following transfection, the cells were washed with warm PBS and exposed to various concentrations of PFOA and PFOS and the positive control phorbol 12-myristate 13-acetate (PMA, Sigma-Aldrich) for 24 h in serum-free medium containing phenol red-free DMEM (PAN Biotech), 1% (v/v) ITS (Capricorn Scientific), 0.1 mg/mL fatty acid-free BSA (Sigma-Aldrich), 100 U/mL penicillin and 100 µg/mL streptomycin. Luciferase activity was analyzed as previously described (Hampf and Gossen 2006). Three individual experiments with four replicate wells per condition were performed.

### CYP7A1 protein determination

Protein expression of CYP7A1 was analyzed via a liquid chromatography–mass spectrometry (LC–MS)-based immunoassay. HepaRG cells were differentiated in 6-well plates and incubated with PFOA, PFOS or 20 µM CSA for 48 h with three replicate wells per condition. After washing the cells with cold PBS, the cells were lysed by adding 300 µL/well lysis buffer (pH 7.2; 1% NP-40, 0.01% sodium dodecyl sulfate (SDS), 0.15 M NaCl, 0.01 M sodium phosphate, 2 mM ethylenediaminetetraacetic acid (EDTA) and 2.5 U/mL benzonase) for 1 h on ice. The protein content was determined using the bicinchoninic acid (BCA) assay (Thermo Fisher Scientific, Waltham, USA). The samples were diluted 1:5, and the BCA assay was performed according to the manufacturer's protocol. The readout was performed with the BioTek ELx808 microplate reader (BioTek, Winooski, USA) at 562 nm. To proteolyze the samples, 150 µg sample was diluted with triethanolamine (TEA; final concentration 50 mM) buffer and denatured at 99 °C. The samples were then reduced by adding tris (2-carboxyethyl) phosphine (TCEP; 5 mM) and alkylated with iodoacetamide (IAA; 10 mM) for 30 min in the dark at room temperature. The tryptic proteolysis (trypsin:protein ratio 1:40) was performed overnight for 16 h at 37 °C with continuous shaking. To stop proteolysis, phenylmethanesulfonyl fluoride (PMSF; 1 mmol/L) was added. The immunoprecipitation following proteolysis was performed as described in (Weiß et al. 2018). For LC–MS analysis, the μ-precolumn (trapping column; 0.3 mm I.D. × 5 mm) and the Acclaim PepMap RSLC C18 (analytical column; 75 μm I.D. × 150 mm; both columns from Thermo Fisher Scientific, Waltham, MA, USA) were used. The separation was achieved using an UltiMate 3000 RSLCnano LC system and the mass spectrometric detection of the analytes using a QExactive™ Plus (both Thermo Fisher Scientific, Waltham, MA, USA). Parallel reaction monitoring (PRM) was used as the measurement method, whereby the duration of the method was 10 min, with the oven temperature set to 55 °C and the flow rate set to 1 µL/min. The data analysis was performed with Skyline 4.2.0. 19072, where the ratio of endogenous to isotopically labeled standard peptide was determined. For the analyte CYP7A1 (UniProtID P22680), the peptide LSSASLNIR was analyzed, whereby only the quantifier ion (y7+; 760.4312) was evaluated for quantification. Lower limit of quantification (LLOQ) was 0.03425 fmol/µg protein.

### Immunolocalization

For the visualization of the bile acid transporter multidrug resistance-associated protein 2 (MRP2), 5 × 10^4^ HepaRG cells/well were seeded on 13-mm coverslips which were previously coated with 0.1% gelatin and differentiated as described before. After treatment with PFOA, PFOS or CSA for 24 h, the cells were washed with PBS and fixed with 4% para-formaldehyde (Thermo Fisher Scientific) diluted in PBS for 20 min at room temperature (RT). The fixed cells were treated with 0.5% Triton-X 100 in PBS for 10 min followed by incubation with 1% BSA in tris-buffered saline containing 0.1% (v/v) Tween 20 (TBST; Sigma-Aldrich) for 1 h at RT. Subsequently, the cells were incubated with primary antibody against MRP2 (1:500 dilution) (#ab172630, Abcam, Cambridge, UK) at 4 °C overnight. After washing with TBST, an incubation with the secondary antibody anti-rabbit Alexafluor633 (1:200 dilution) (Life Technologies, Carlsbad, USA) was performed for 1 h at RT. Finally, the cells were washed again with TBST and nuclei were stained with DAPI (Thermo Fisher Scientific) for 20 min at RT. The coverslips were washed with PBS and covered with Vectashield HardSet antifade mounting medium (Vector Laboratories, Burlingame, USA) on microscope slides. Immunofluorescence images were obtained by Cell Discoverer 7 (Zeiss, Oberkochen, Germany). Three individual experiments were performed.

### Transporter functionality

The transporter activity of MRP2 was measured through determination of the fluorescence intensity of 5(6)-carboxy-2′,7′-dichlorofluorescein (CDF) in HepaRG cell layer. Therefore, HepaRG cells were differentiated in 24-well plates and treated with PFOA, PFOS or CSA for 24 h. After treatment, the cells were incubated with 3 µM 5(6)-carboxy-2′,7′-dichlorofluorescein-diacetate (CDFDA, Sigma-Aldrich) in phenol red-free William’s E medium without supplements for 30 min at 37 °C. CDFDA passively enters the cell and gets hydrolyzed by intracellular esterases to the fluorescent dye CDF, a substrate for MRP2. After washing, nuclei were labeled using Hoechst33342 (5 µg/mL in PBS) (Thermo Fisher Scientific). Imaging was done with the Cell Discoverer 7 by scanning the well automatically (eight images per well) at 10× magnification in four individual experiments. The quantification of fluorescence intensity was conducted using Zeiss Zen Imaging Software, and a threshold (2.000 RFU) was set to exclude unspecific fluorescence signals, which was equal for the analysis of each image.

### Cytoskeleton distribution

The distribution of the cytoskeleton was visualized by staining of F-actin and the tight junction protein Zonula Occludens-1 (ZO-1). HepaRG cells were differentiated in 24-well plates and treated with different concentrations of PFOA or PFOS, or with 20 µM CSA for 24 h. The cells were fixated with ice-cold methanol for 20 min at RT for ZO-1 staining and with 4% para-formaldehyde for 20 min at RT for F-actin staining followed by incubation with 0.5% Triton-X 100 in PBS for 10 min at RT. Subsequently, the cells were treated with 1% BSA in TBST for 1 h at RT. After washing with TBST, ZO-1 (1:400 dilution) (#13663, Cell signaling, Frankfurt am Main, Germany) was stained for 4 h at RT and F-actin (2 drops/mL) (#R37110, Thermo Fisher Scientific) was stained for 30 min at RT. After washing with TBST, nuclei were labeled with DAPI (Thermo Fisher Scientific) for 20 min at RT. Imaging was conducted with the Cell Discoverer 7.

### Measurement of cholesterol content

Total cholesterol content in medium and in HepaRG cells was quantified using the AmplexRed Cholesterol Assay (Thermo Fisher Scientific) according to the manufacturer’s protocol. Briefly, HepaRG cells were differentiated in 12-well plates. After treatment with different concentrations of PFOA or PFOS or 20 µM CSA for 24 h and 48 h, the cells were placed on ice and the medium was collected. The cells were washed with cold PBS and lysed with RIPA buffer (pH 7.5; 50 mM Tris–HCl, 150 mM NaCl, 2 μM EGTA, 0.1% sodium dodecyl sulfate (SDS), 0.5% deoxycholic acid) containing a protease inhibitor cocktail (Complete Protease Inhibitor Cocktail Tablets, Roche, Mannheim, Germany) for 10 min on ice. Cell lysate was collected and sonicated (10 s, 25% amplitude, 2 × pulsation). Cell lysates and cell culture supernatants were stored at − 80 °C until measurement. Total cholesterol content in supernatants and cells was determined via AmplexRed Cholesterol Assay against a cholesterol standard curve. Therefore, 50 µL of the supernatant (undiluted) or the cell lysate (1:5 dilution in reaction buffer) was mixed with 50 µL of AmplexRed working solution in a flat-buttoned black 96-well plate and incubated for 30 min at 37 °C. The fluorescence (540 nm ex, 590 nm em) was measured with a Tecan infinite M200 PRO microplate reader (Tecan Group Ltd., Männedorf, Switzerland). In each experiment, 10 µM of hydrogen peroxide served as a positive control for the assay and a no-cholesterol control was used to correct background signals. For normalization, the protein amount of each sample was determined via Bio-Rad Protein Assay (Biorad) according to the manufacturer’s protocol against a BSA standard curve. Three individual experiments were performed.

### Measurement of endogenous bile acid content

HepaRG cells were differentiated in 6-well plates and treated with various concentrations of PFOA or PFOS or 20 µM CSA for 48 h. Three replicate wells were treated per condition. After treatment, the cells were placed on ice and the medium was collected. After washing with cold PBS, the cells were incubated with Trypsin/EDTA (Capricorn Scientific) for 25 min at 37 °C. The cells were collected, washed again with cold PBS and samples with same treatment condition were pooled. After centrifugation, PBS was aspirated and the cell pellets and the cell culture supernatants were stored at − 20 °C until measurement. Protein amount was determined via the Bio-Rad Protein Assay as described before. Three individual experiments were performed. Bile acid analysis was made using ultra-performance liquid chromatography (UPLC) coupled to tandem mass spectrometry according to previous work (Tremaroli et al. 2015). Briefly, cells and media were extracted by adding methanol, which contained deuterated internal standards. After vortex and centrifugation, the methanol was evaporated under a stream of nitrogen and the samples were reconstituted in methanol:water 1:1 (v/v) and injected onto the UPLC system (Infinity1290, Agilent Technologies, Palo Alto, CA, USA). Separation was made on a Kinetex C18 column (Phenomenex, Torrance, CA, USA) using water and acetonitrile as mobile phases. Detection was made in negative mode using a QTRAP 5500 mass spectrometer (AB Sciex, Concord, Canada).

### Statistical analysis

For determination of differences between control and treatment groups, one-way analysis of variance (ANOVA) was performed with a Dunnett’s post hoc test. Data were considered significantly different when *p < 0.05, **p < 0.01 or ***p < 0.001.

## Results

### Cytotoxic potential

**Fig. 1:**
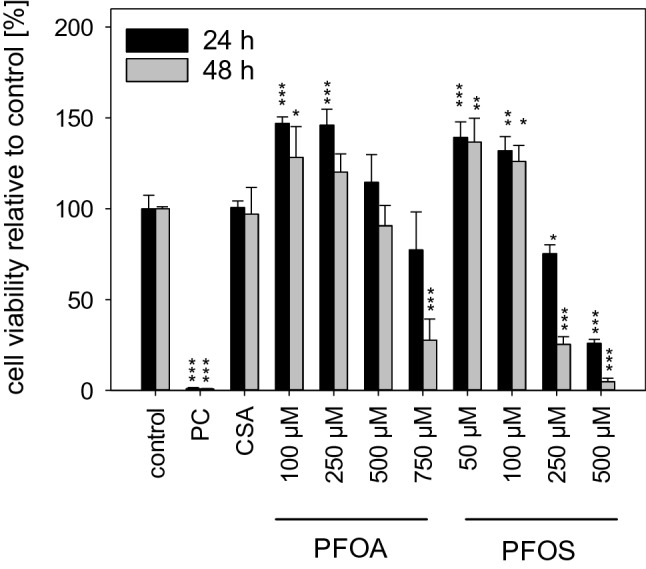
Cell viability of HepaRG cells. The cells were exposed to various concentrations of PFOA or PFOS, or to 20 µM CSA, for 24 h or 48 h, and cellular viability was measured using the MTT assay. Viability is represented as percentage, compared to untreated cells (control) set to 100%. In each experiment, Triton X-100 (0.01%) served as positive control (PC). Data are presented as mean + SD. ***p < 0.001, **p < 0.01, *p < 0.05; one-way ANOVA with Dunnett’s post hoc test

Prior to the analysis of molecular effects, non-cytotoxic concentrations of PFOA and PFOS were determined via MTT assay. Both compounds were non-cytotoxic in differentiated HepaRG cells cultured in serum-free medium up to concentrations of 500 µM and 100 µM, respectively (Fig. 1). Even though this applies to incubation for both 24 h and 48 h, it was decided to use these concentrations as the highest test concentrations only for experiments in which the cells were exposed to the substances for 24 h. For the experiments with an incubation time of 48 h, lower maximal PFOA and PFOS concentrations of 250 µM and 50 µM, respectively, were used. CSA, used at 20 µM in subsequent experiments as a cholestasis-inducing model compound, was non-cytotoxic at that concentration in HepaRG cells (Fig. 1).

### Gene expression analysis

**Fig. 2:**
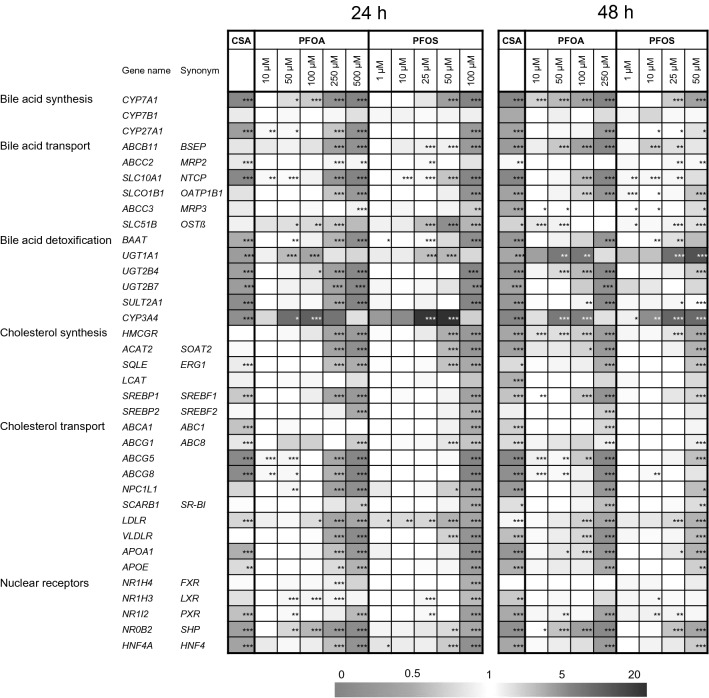
Gene expression analysis of genes related to cholesterol homeostasis. HepaRG cells were exposed to various concentrations of PFOA or PFOS, or to 20 µM CSA, for 24 h or 48 h. Untreated cells served as control. mRNA levels were normalized to *GAPDH* expression. Fold changes relative to untreated cells (mean of three individual experiments) are presented in the heat map. ***p < 0.001, **p < 0.01, *p < 0.05; one-way ANOVA with Dunnett’s post hoc test

To examine whether PFOA and PFOS have an impact on the expression of genes whose products are involved in synthesis, transport or metabolism of cholesterol and bile acids, HepaRG cells were incubated for either 24 h or 48 h with different non-toxic concentrations of PFOA or PFOS. The results of the subsequent gene expression analysis are presented as a heatmap in Fig. 2. Notably, most of the genes examined in this study were similarly downregulated by both PFOA and PFOS, indicating that both substances broadly affect cellular functions associated with cholesterol homeostasis. There were, however, a few exceptions where upregulation was observed; expression of OSTβ *(SLC51B)*, a basolateral bile acid efflux transporter, *ABCG1*, a basolateral cholesterol efflux transporter, *UGT1A1*, an UDP-glucuronosyltransferase required for the elimination of bilirubin, and *CYP3A4*, a phase I enzyme that is amongst other functions able to detoxify bile acids by hydroxylation, were strongly upregulated by PFOA and PFOS. All effects on gene expression were observed at both incubation times, and in most cases, the effects were more pronounced after 48 h of incubation, as compared to 24 h. Of note, incubation of the cells with 20 µM CSA resulted in similar effects on gene expression as the incubation with high concentrations of PFOA or PFOS. Only three divergent effects were observed; CSA inhibited gene expression of the PFOA- and PFOS-induced genes *UGT1A1* and *CYP3A4*, and CSA slightly induced gene expression of *LDLR*, which was inhibited by PFOA and PFOA (Fig. 2). As CSA is known to induce cholestasis in vivo (Lee 2003; Padda et al. 2011), the comparable gene expression patterns may indicate a cholestatic potential of PFOA and PFOS at high concentrations.

### Reporter gene assay and protein level of CYP7A1

**Fig. 3:**
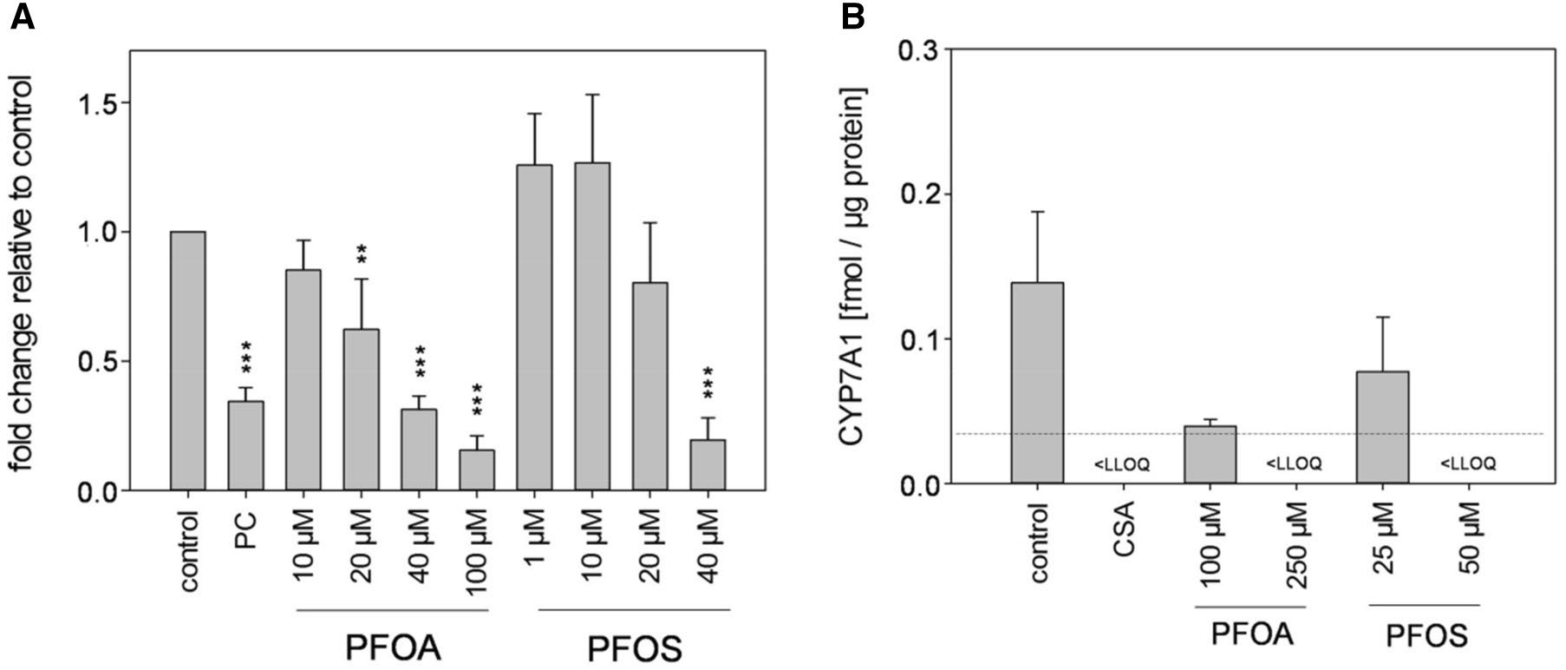
Promotor activity and protein level of CYP7A1. **a** HepG2 cells were transfected with the promotor plasmid pGL4.14–CYP7A1-Prom and the *Renilla*-luciferase construct pcDNA3-Rluc. Luciferase activity was measured after 24-h exposure to various concentrations of PFOA or PFOS. Transfected cells treated with 1 µM phorbol 12-myristate 13-acetate (PMA) served as a positive control (PC). Values were normalized to *Renilla reniformis* luciferase activities and compared to untreated cells (control). Data are represented as mean + SD. ***p < 0.001, **p < 0.01; one-way ANOVA with Dunnett’s post hoc test. **b** HepaRG cells were exposed to various concentrations of PFOA or PFOS, or to 20 µM CSA for 48 h. CYP7A1 protein was analyzed via mass spectrometry-based immunoassay. The dashed line indicates the lower limit of quantification (LLOQ) for this protein quantification method. Data are presented as mean + SD relative to untreated cells (control) from three replicate wells per condition

CYP7A1 is the key enzyme that catalyzes the initial step in cholesterol catabolism and bile acid synthesis, and a deficiency of CYP7A1 is associated with hypercholesterolemia (Pullinger et al. 2002). Since gene expression analysis has revealed that *CYP7A1* was strongly downregulated by PFOA and PFOS (Fig. 2), we further examined the impact of both substances on the promoter activity of *CYP7A1* via luciferase-based reporter gene assays. Incubation of cells transfected with the reporter plasmid for *CYP7A1* with increasing concentrations of PFOA and PFOS correlated with a decrease in luciferase activity indicating an inhibitory effect of PFOA and PFOS on the activity of the *CYP7A1* gene (Fig. 3a). In addition, we examined whether the inhibition of *CYP7A1* gene expression as well as the inhibition of *CYP7A1* promoter activity by PFOA and PFOS is also reflected at the protein level. In comparison to the untreated control, incubation of HepaRG cells with PFOA or PFOS for 48 h clearly decreased the protein amount of CYP7A1 (Fig. 3b). Thus, PFOA and PFOS had a strong impact on CYP7A1 both at the protein level and at the level of gene expression.

### Cholesterol and bile acid profiles

**Fig. 4:**
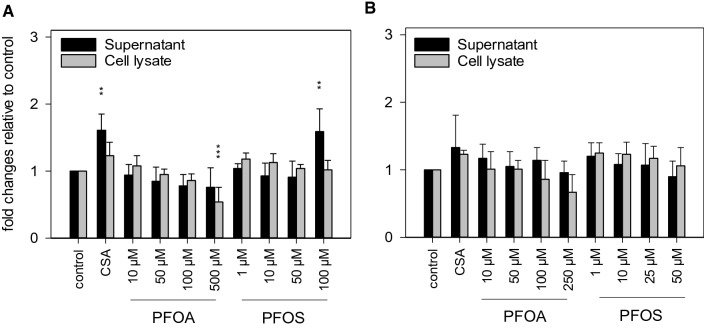
Cholesterol content in cell lysates and cell culture supernatants. HepaRG cells were treated with various concentrations of PFOA or PFOS, or 20 µM CSA, for (**a**) or 48 h (**b**). Cholesterol levels in cell lysates and supernatants were quantified using the fluorescence-based AmplexRed Cholesterol Assay. Data are presented as mean + SD. ***p < 0.001, **p < 0.01; one-way ANOVA with Dunnett’s post hoc test

To examine the impact of PFOA and PFOS on cholesterol and bile acid metabolism in HepaRG cells in more detail, the content of cholesterol and of selected bile acids was determined in cellular lysates as well as in cell culture supernatants. These experiments were again carried out in the absence of serum, because serum contains considerable amounts of cholesterol and of different bile acids. Cholesterol was quantified using the fluorescence-based AmplexRed Cholesterol Assay. Total cholesterol was hardly affected by PFOA and PFOS. A 1.6-fold increase was observed in the cell culture supernatant after incubation of HepaRG cells with CSA or with 100 µM PFOS for 24 h. Incubation with PFOA, on the other hand, resulted in an intracellular decrease of total cholesterol to a level of about 50%, as compared to untreated cells (Fig. 4a). These effects were no longer observed when the cells were exposed to the test compounds for 48 h, pointing to mechanisms that account for balancing of cholesterol levels in HepaRG cells after substance-induced deregulation.

**Fig. 5:**
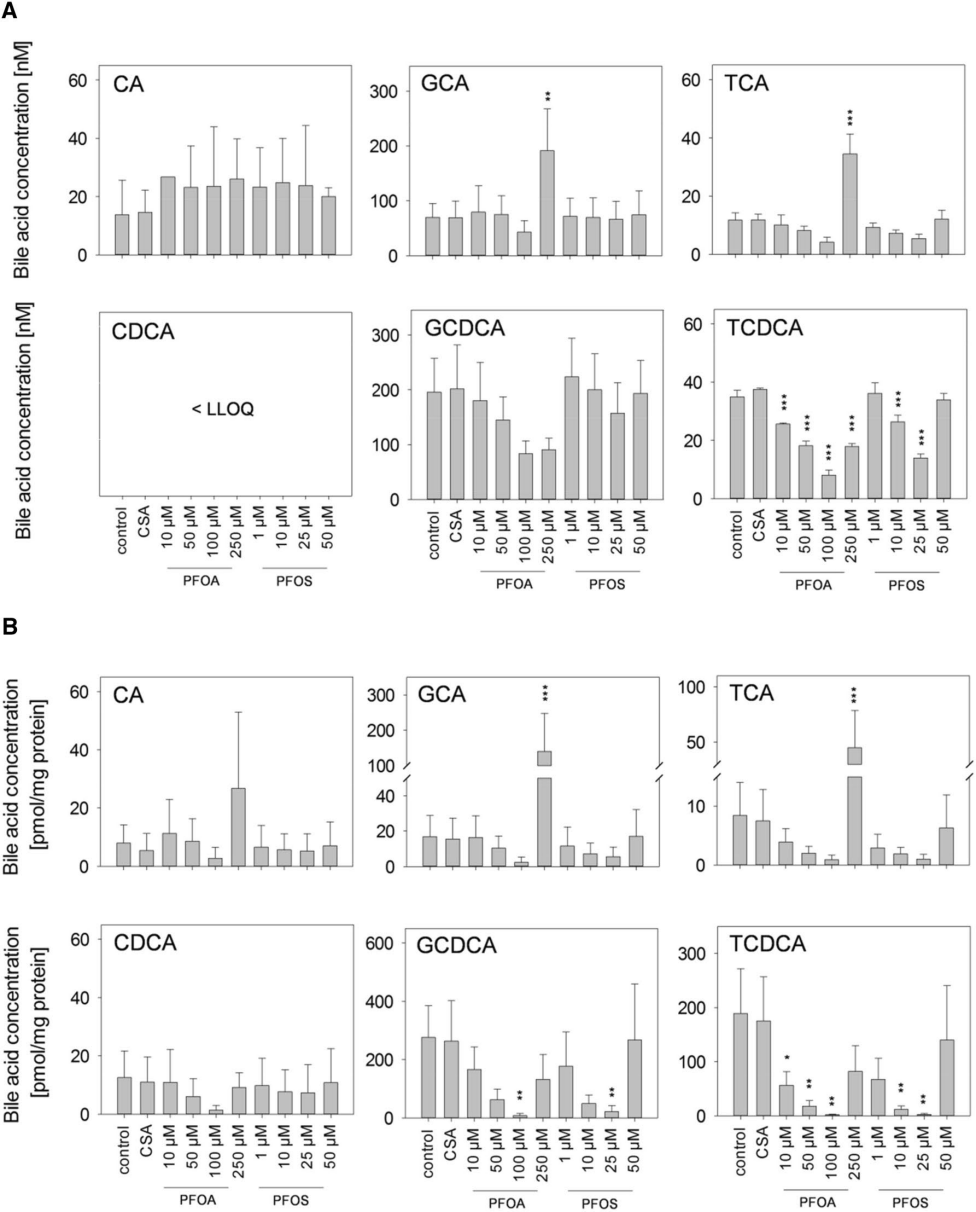
Bile acid profiles in cell lysates and cell culture supernatants. HepaRG cells were treated with the indicated concentrations of PFOA or PFOS, or with 20 µM CSA, for 48 h. Quantification of bile acids was conducted using ultra performance liquid chromatography (UPLC) coupled to tandem mass spectrometry. Data are presented as mean + SD out of three biological replicates with three replicate wells per condition. ***p < 0.001, **p < 0.01, *p < 0.05; one-way ANOVA with Dunnett’s post hoc test

Bile acids are produced from their precursor cholesterol, and alterations in bile acid profiles could be an indicator for the onset of cholestasis. Therefore, we examined whether the treatment of HepaRG cells with PFOA or PFOS would result in alterations in the intra- and extracellular levels of selected bile acids. The content of cholic acid (CA) in the cell culture supernatants was slightly increased after 48 h of incubation; however, these elevated CA levels were not statistically significant (Fig. 5a). Extracellular chenodeoxycholic acid (CDCA) levels were below the LLOQ of 2 nM. In the case of the glycine and taurine conjugates of these two bile acids, a concentration-dependent decrease was observed in the supernatants for the incubation with PFOA and PFOS, except for the highest test concentrations of 250 µM PFOA and 50 µM PFOS. For these concentrations, the observed decrease was inversed resulting in levels comparable to the levels observed in untreated control cells (as, e.g., seen for taurocholic acid (TCA) and taurochenodeoxycholic (TCDCA) with 50 µM PFOS) or even in levels that significantly exceeded the levels in untreated control cells (as, e.g., seen for glycocholic acid (GCA) and TCA with 250 µM PFOA) (Fig. 5a). These PFOA- and PFOS-mediated effects on the conjugated bile acids were also observed and even more pronounced in cell lysates which may indicate that the extracellular content of these conjugated bile acids simply reflects intracellular alterations, and that transport processes such as import and/or export of the conjugated bile acids are possibly hardly affected (Fig. 5b). Regarding the non-conjugated bile acids, the intracellular contents of CA and CDCA were not affected by PFOS; whereas, they were deregulated by PFOA in a way similar to their glycine and taurine conjugates (Fig. 5b). Taken together, the bile acid profiles in HepaRG cells were strongly affected by PFOA and PFOS, and different mechanisms may account for the concentration-dependent effects that finally resulted in partially biphasic responses as observed in our analysis.

### Impairment of bile canaliculi morphology and bile flow

**Fig. 6:**
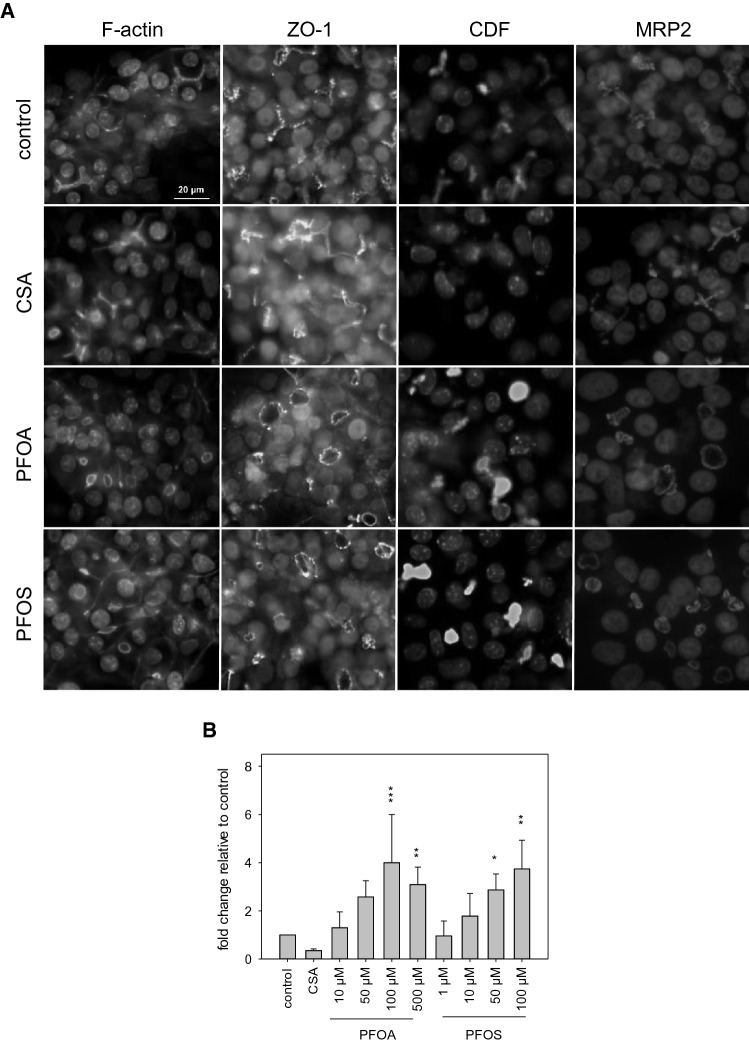
Effects of PFOA and PFOS on the distribution of cytoskeletal proteins and on bile flow. HepaRG cells were treated with various concentrations of PFOA or PFOS, or with 20 µM CSA. Immunostaining was performed using antibodies directed against F-actin, ZO-1 and MRP2 together with the respective secondary antibody (F-actin = green, ZO-1 = yellow, MRP2 = red). Nuclei were stained with DAPI (blue). CDF accumulation (green) in bile canaliculi was quantified; nuclei were stained with Hoechst33342 (blue). **a** Representative microscopic images for untreated control cells and for the treatments with 20 µM CSA, 100 µM PFOA, or 50 µM PFOS. **b** Quantification of CDF fluorescence intensity. Fold changes relative to untreated cells (mean + SD of four individual experiments) are presented. ***p < 0.001, **p < 0.01, *p < 0.05; one-way ANOVA with Dunnett’s post hoc test (color figure online)

Differentiated HepaRG cells form polarized structures and functional bile canaliculi, and, thus, are a good model for investigating the morphology of bile canaliculi which is essential for maintaining bile flow. Therefore, HepaRG cells were exposed to PFOA and PFOS and subsequently subjected to immunostaining. Bile canaliculi can be visualized by immunostaining of the actin cytoskeleton, tight-junctional proteins such as ZO-1 and transporters which are localized in the canalicular membrane, such as the bile acid efflux transporter MRP2. Fluorescence imaging revealed the typical, tubular Y-shaped form of bile canaliculi in untreated HepaRG cells. After treatment with PFOA or PFOS, the Y-shape of bile canaliculi shifted to an O-shaped form indicating an increase in bile lumen and, therefore, a dilation of the bile canaliculi by PFOA and PFOS (Fig. 6a). In contrast, CSA treatment led to a slight constriction of bile canaliculi resulting in a decrease of bile lumen.

To examine whether the observed bile canaliculi deformation by PFOA and PFOS has an impact on bile flow, we took advantage of the fluorophore CDFDA. CDFDA can be hydrolyzed to its fluorescent diacetate CDF which is subsequently transported via MRP2 inside the bile canaliculi. Untreated control cells showed that CDF was exclusively located inside the bile canaliculi; whereas, CDF was barely found in the canaliculi of CSA-treated samples indicating that CSA inhibits either CDFDA hydrolysis or CDF transport by MRP2. Representative microscopic images of accumulated CDF are shown in Fig. 6a. Treatment of the cells with either PFOA or PFOS resulted in a strong increase in fluorescence intensity (Fig. 6a). The increase of fluorescence intensity occurred in a concentration-dependent manner with a maximal fourfold increase at either 100 µM PFOA or 50 µM PFOS (Fig. 6b). The observed increased amounts of CDF in the bile canaliculi-like structures may be due to a more efficient intracellular hydrolysis of CDFDA, a more efficient transport of CDF by MRP2, or may simply reflect the larger lumina of these structures which should be proportional to the CDF-related fluorescence intensity.

## Discussion

Based on epidemiological data, EFSA has identified elevated total cholesterol blood serum levels as the most critical effect induced by PFOA and PFOS in humans (EFSA et al. 2018). As this observation is opposed to results obtained from animal studies, additional experimental data are required to elucidate the underlying molecular mechanisms that are responsible for the observed species differences regarding the PFOA/PFOS-mediated effects on cholesterol homeostasis.

In the present study, we used the cell line HepaRG as a model for human hepatocytes. In HepaRG cells, cholesterol and bile acid metabolism is very similar to primary hepatocytes based on the localization and functional activity of sinusoidal and canalicular transporters (Bachour-El Azzi et al. 2015; Le Vee et al. 2006, 2013; Antherieu et al. 2010). Moreover, the expression of CYP enzymes and nuclear receptors involved in bile acid metabolism is close to the pattern of human primary hepatocytes (Rogue et al. 2012). The cell line has already been used to study drug-induced liver injury such as cholestasis (Anthérieu et al. 2013; Rodrigues et al. 2018; Sharanek et al. 2015, 2016). With a focus on cholesterol and bile acid synthesis and metabolism, all experiments were conducted in the absence of serum. Thus, cholesterol and bile acid metabolism was exclusively dependent on de novo synthesis and cellular metabolism.

In this study, the cholesterol content in medium and cells was barely affected by PFOA and PFOS suggesting that both substances did not impair the de novo synthesis of cholesterol and/or that cholesterol levels are tightly regulated in HepaRG cells. As cholesterol is the precursor for bile acid synthesis, we examined the profiles of selected bile acids after treatment of cells with PFOA and PFOS. Both compounds had substantial impact on the bile acid content in the cells and in the cell culture supernatants. The observed strong alterations in bile acid levels were not reflected by concomitant changes at the cholesterol level, which might be due to the fact that cholesterol levels in the cells (nmol/mg protein range) are several orders of magnitude above the levels of the different bile acids (pmol/mg protein range). Thus, bile acid levels are strongly affected by external stimuli such as PFAS, while these alterations are not visible at the cholesterol level. Regarding intracellular bile acid contents, we have observed a dose-dependent decrease of selected bile acids followed by a renewed bile acid synthesis at the highest test concentration of PFOA and PFOS. The observed alterations in intracellular bile acid contents point to an at least biphasic pattern that might be due to different overlapping regulatory mechanisms such as feedback loops being involved in the regulation of intracellular bile acid contents. This hypothesis, however, has to be examined in detail in further studies that should focus—in addition to dose-dependent alteration—also on time-dependent effects of PFOA and PFOS on bile acid synthesis. As an example, CSA, which is known to induce cholestasis in vivo (Lee 2003; Padda et al. 2011), showed no impact on the bile acid profiles under our test conditions. Sharanek et al. (2015) investigated the time-dependent synthesis of endogenous bile acids in CSA-treated HepaRG cells without serum. They showed an intracellular accumulation of bile acids after 4-h treatment with CSA; whereas, this effect was no longer observed after 24-h treatment. In the present study, bile acid content was measured after 48-h incubation indicating that CSA seems to have only short-term effects on de novo bile acid synthesis.

The first- and rate-limiting step for the classic synthesis of bile acids from cholesterol is catalyzed by CYP7A1. Gene expression analysis as well as reporter gene assays revealed an inhibitory effect of PFOA and PFOS on *CYP7A1* at the transcriptional level. Moreover, CYP7A1 was also strongly decreased at the protein level. The inhibitory effect of PFOA and PFOS on CYP7A1 might be the molecular initiating event leading to the observed alterations in bile acid profiles.

After synthesis, most of the bile acids are immediately conjugated to increase their solubility which is important for efficient transport and detoxification. Conjugation of the parent bile acids CA and CDCA with taurine and glycine is mainly mediated by bile acid amino acid transferase (*BAAT*). Other routes of conjugation are sulfation by SULT2A1 and glucuronidation via UGTs such as UGT2B4 (Alnouti 2009). Gene expression revealed that all three genes were downregulated by PFOA and PFOS indicating an inhibitory effect on the conjugation routes and, therefore, detoxification and excretion of bile acids. A protective mechanism against the accumulation of toxic bile acids is the upregulation of *CYP3A4* (Chen et al. 2014). CYP3A4 hydroxylates bile acids not only to increase their hydrophilicity, but also to increase their conjugation (Li and Apte 2015) which exerts an essential protective mechanism in cholestasis in vivo. Indeed, *CYP3A4* gene expression was strongly upregulated by PFOA and PFOS already at low concentrations indicating that CYP3A4 might be activated in HepaRG cells as an alternative detoxification route for bile acids. Interestingly, neither PFOA nor PFOS activated human PXR in a luciferase-based reporter gene assay (Behr et al. 2020), indicating that mechanisms other than PXR activation may account for PFOA/PFOS-mediated induction of *CYP3A4* gene expression.

According to the adverse outcome pathway (AOP) for cholestasis (Vinken et al. 2013), risk of bile acid accumulation is compensated via the activation of several nuclear receptors such as FXR, PXR or CAR. FXR plays a central role in the prevention of hepatocellular injury due to elevated bile acids by increasing the expression of genes involved in bile acid export (*SLC51B*, *ABCC2*, *ABCB11*) or bile acid conjugation (*CYP3A4*, *UGT2B4*). Moreover, FXR activation leads to the transcriptional activation of SHP (*NR0B2*), a nuclear receptor factor that in turn results in the repression of several genes involved in bile acid uptake (*SLC10A1*, *SLCO1B1*) and in particular of *CYP7A1*, the key enzyme of bile acid synthesis. In our experiments, expression of *CYP3A4*, *UGT1A1*, *SLC51B* and *ABCC2* was increased; whereas, *UGT2B4, UGT2B7*, *SLC10A1*, *ABCB11* and *CYP7A1* gene expression was decreased by PFOA and PFOS. Our qPCR results may indicate that PFOA and PFOS treatment leads to an activation of FXR. However, we have recently observed that PFAS including PFOA and PFOS do not directly activate FXR in a transactivation assay (Behr et al. 2020). However, qPCR revealed a downregulation of *NR0B2* expression by PFOA and PFOS which would indicate that the inhibition of the transcriptional activity of *CYP7A1* by PFOA and PFOS is most likely mediated by FXR–SHP-independent pathways. A previous study with data obtained from human liver indicated that in case of upregulated bile acid synthesis, the repression of *CYP7A1* gene expression is predominantly mediated by SHP-independent pathways involving, e.g., HNF4α (Abrahamsson et al. 2005). HNF4α is a constitutively active nuclear receptor and is important for the transcriptional activity of genes encoding for several key enzymes of bile acid synthesis such as CYP7A1, CYP27A1, CYP8B1 or BAAT (Crestani et al. 1998; Chiang 2009; Abrahamsson et al. 2005; Garuti et al. 2002; Chen and Chiang 2003) as well as bile acid transport proteins such as NTCP, OATP1B1, MRP2 and BSEP (Jung and Kullak-Ublick 2003; Davis et al. 2002; Svoboda et al. 2011; Kamiyama et al. 2007). In the present study, *HNF4A* gene expression was strongly downregulated by PFOA and PFOS treatment. PFOA/PFOS-mediated repression of *HNF4A* could in turn result in the observed repression of *CYP7A1* and also of most of the other genes mentioned above. Moreover, previous studies have shown that PFOA and PFOS are able to decrease HNF4α at the protein level in the human hepatoma cell line HepG2 (Beggs et al. 2016; Scharmach et al. 2012). Thus, PFOA/PFOS-mediated inhibition of HNF4α either at the protein level and/or at the level of gene transcription may be the key event that finally leads to the observed effects in HepaRG cells in the present study that may account for the onset of cholestasis and that may also be related to the elevated blood serum cholesterol levels observed in humans in vivo.

All in all, the observed alterations in gene expression as well as the decrease of bile acids by PFOA and PFOS point towards a feedback loop to prevent bile acid accumulation. Bile acid accumulation, which further progresses to cholestasis, could be mediated by an altered bile flow, disruption of cholesterol and bile acid formation, or disruption of bile canalicular morphology. Vinken et al. (2013) assumed that BSEP inhibition is the most likely reason for the onset of cholestasis as several cholestatic drugs inhibit BSEP. In our experiments, we have seen downregulation of *ABCB11* gene expression by PFOA and PFOS which indicates reduced amounts of BSEP resulting in consequences that would be similar to an inhibition of BSEP activity. Reduced bile flow along with bile acid accumulation inside the cells or within the bile canaliculi could also be a result of, e.g., a compromised cytoskeleton or a disruption of tight junctions. Indeed, in the present study, we have shown a dilatation of bile canaliculi by PFOA or PFOS as identified by immunostaining. These experiments revealed a disorganization of the actin cytoskeleton and a structural redistribution of the tight-junctional protein ZO-1 by PFOA and PFOS. Notably, comparable morphological changes have been observed in cholestatic livers in vivo (Jansen et al. 2017; Ghallab et al. 2019). Thus, for the first time, we have shown that PFOA and PFOS exert effects in hepatocytes that result in morphological changes of structures that might be of relevance also for the in vivo situation. The observed disorder of bile canalicular morphology might result in an impairment of bile flow which was representatively investigated using CDF as a model compound. PFOA and PFOS led to an enhanced secretion of CDF, as visualized by increased fluorescence intensity in the bile canaliculi. As CDF is a specific substrate for MRP2, the enhanced secretion could be attributed to an increased MRP2 activity. This could be a protective feedback mechanism for the dilated bile canaliculi to maintain bile acid flow. CSA inhibited the secretion of CDF which could be attributed to the constriction of bile canaliculi after cell treatment with CSA, an effect which has recently been reported by several groups (Sharanek et al. 2014, 2015, 2016

In conclusion, we have shown using the HepaRG cell line as a model for human hepatocytes that PFOA and PFOS affect bile acid metabolism at the transcriptional and at the metabolite level. Our results are in line with the assumption that PFOA and PFOS induce dilatation of bile canaliculi which enhance the risk for bile acid accumulation resulting in the initiation of a feedback loop including FXR-dependent and FXR-independent pathways to prevent cells from injury. From our in vitro data, it can be concluded that concentrations of PFOA or PFOS higher than 10 µM may contribute to the development of cholestasis in vivo. It has to be noted, however, that the concentrations of PFOA and PFOS used in our study are three–four orders of magnitude above the blood serum level of the common Western population (Kato et al. 2011; Calafat et al. 2007).

## Electronic supplementary material

Below is the link to the electronic supplementary material.

Supplementary file1 (DOCX 17 kb)

## Abbreviations

BA: Bile acid
BC: Bile canaliculi
BSA: Bovine serum albumin
BSEP: Bile salt export pump
CA: Cholic acid
CDCA: Chenodeoxycholic acid
CDF: 5(6)-Carboxy-2′,7′-dichlorofluorescein
CDFDA: 5(6)-Carboxy-2′,7′-dichlorofluorescein-diacetate
CSA: Cyclosporine A
DMSO: Dimethyl sulfoxide
FBS: Fetal bovine serum
GCA: Glycocholic acid
GCDCA: Glycochenodeoxycholic acid
HBSS: Hank’s balances salt solution
ITS: Insulin-transferrin-selenium
MRP2: Multidrug resistance-associated protein 2
MTT: 3-(4,5-Dimethylthiazol-2-yl)-2,5-diphenyltetrazolium bromide
NTCP: Na^+^-taurocholate cotransporting polypeptide
PBS: Phosphate buffered saline
PFOA: Perfluorooctanoic acid
PFOS: Perfluorooctanesulfonic acid
qPCR: Quantitative polymerase chain reaction
PFAS: Perfluoroalkylated substances
RT: Room temperature
TBST: Tris-buffered saline with 0.1% (v/v) Tween 20
TCA: Taurocholic acid
TCDCA: Taurochenodeoxycholic acid
THCA: Tauronor-nor-24-DBD
ZO-1: Zonula occludens-1
