# Correction to: Impairment of bile acid metabolism by perfluorooctanoic acid (PFOA) and perfluorooctanesulfonic acid (PFOS) in human HepaRG hepatoma cells

**DOI:** 10.1007/s00204-021-03089-x

**Published:** 2021-06-29

**Authors:** Anne‑Cathrin Behr, Anna Kwiatkowski, Marcus Stahlman, Felix Florian Schmidt, Claudia Luckert, Albert Braeuning, Thorsten Buhrke

**Affiliations:** 1 Department of Food Safety, German Federal Institute for Risk Assessment, Max‑Dohrn‑Str. 8‑10, 10589 Berlin, Germany; 2 Wallenberg Laboratory, Department of Molecular and Clinical Medicine, Sahlgrenska Academy, Gothenburg University, 413 45 Gothenburg, Sweden; 3 Signatope GmbH, 72770 Reutlingen, Germany

## Correction to: Archives of Toxicology (2020) 94:1673–1686 10.1007/s00204-020-02732-3

The article “Impairment of bile acid metabolism by perfluorooctanoic acid (PFOA) and perfluorooctanesulfonic acid (PFOS) in human HepaRG hepatoma cells”, written by Anne-Cathrin Behr, Anna Kwiatkowski, Marcus Ståhlman, Felix Florian Schmidt, Claudia Luckert, Albert Braeuning and Thorsten Buhrke, was originally published Online First without Open Access. After publication in volume 94, issue 5, page 1673–1686 the author decided to opt for Open Choice and to make the article an Open Access publication. Therefore, the copyright of the article has been changed to © The Author(s) 2020 and the article is forthwith distributed under the terms of the Creative Commons Attribution 4.0 International License, which permits use, sharing, adaptation, distribution and reproduction in any medium or format, as long as you give appropriate credit to the original author(s) and the source, provide a link to the Creative Commons licence, and indicate if changes were made. The images or other third party material in this article are included in the article’s Creative Commons licence, unless indicated otherwise in a credit line to the material. If material is not included in the article’s Creative Commons licence and your intended use is not permitted by statutory regulation or exceeds the permitted use, you will need to obtain permission directly from the copyright holder. To view a copy of this licence, visit http://creativecommons.org/licenses/by/4.0.

Open access funding enabled and organized by Projekt DEAL.

Original article has been corrected.

