# Supplementary material for: Impairment of bile acid metabolism by perfluorooctanoic acid (PFOA) and perfluorooctanesulfonic acid (PFOS) in human HepaRG hepatoma cells
Source: Arch Toxicol. 2020 Apr 6;94(5):1673–86. doi: 10.1007/s00204-020-02732-3 (PMC8241792; doi:10.1007/s00204-020-02732-3)
Supplement: Supplementary file 1 — Supplementary file1 (DOCX 17 kb) [file 204_2020_2732_MOESM1_ESM.docx]

Table S 1: Primer sequences used in this study

|  | Primer sequence (5´- 3´) | |
| --- | --- | --- |
|  | forward | reverse |
| *ABCA1* | CCTACAGTGATCCCAGCGTG | GGCAGGTACAGCGTGAAGTA |
| *ABCB11* | TAGCCCTGGAGCATTGACAA | CATGGCCACAGTGACGTTAG |
| *ABCC2* | CAATATCCTGCGCTTTCCCC | CAAGTCATCCCCTCCCAAGT |
| *ABCC3* | CCGCATCCTGGTTTTAGACG | CAGGTATCAAACTGGGTGCG |
| *ABCG1* | CGCCTCATTGCCTATTTTGT | CCTCGGCCACAGTGTCTAAT |
| *ABCG5* | TCCTGAGGTTGCCCGATTTG | ATGGACAGCAGAGCCACTAC |
| *ABCG8* | AAACTTGAGCAGCCTGTGGA | GATGGTGAGGTTCCCGAGAG |
| *ACAT2* | ACTTGGCTTACTTGAGAACAGGA | CAGTCAGTGGCATCTCACCTA |
| *APOA1* | GTGACCTCCACCTTCAGCA | CCAGATCCTTGCTCATCTCC |
| *APOE* | TGATGGACGAGACCATGAAG | TGTTCCTCCAGTTCCGATTT |
| *BAAT* | CCTCATGGCTTGGTACTG | GAGGAGGTGCCACACACTT |
| *CYP27A1* | TTCGAGAAACGCATTGGCTG | GGAGGAAGGTGGCATAGAGTG |
| *CYP3A4* | TCACAAACCGGAGGCCTTTT | TGGTGAAGGTTGGAGACAGC |
| *CYP7A1* | GACACACCTCGTGGTCCTCT | TTTCATTGCTTCTGGGTTCC |
| *CYP7B1* | TCAAAGCAGGCAAGATGTCCT | CCACAGAGGCCCAGAGAAAG |
| *HMGCR* | GACGTGAACCTATGCTGGTCAG | GGTATCTGTTTCAGCCACTAAGG |
| *HNF4A* | AGGACTACATCAACGACCGC | ATCTGCTCGATCATCTGCCA |
| *LCAT* | CTCGGCTGTCTACACTTGCT | GCCATCAATAAAGCGGTCCT |
| *LDLR* | GCTCCATCGCCTACCTCTTC | TTCTATTGCTGGCCACCTCC |
| *NPC1L1* | GTCGTCACCTTGGGACTCAT | CTGGACATCACCTTCCACCT |
| *NR0B2* | AGGGACCATCCTCTTCAACC | TCCAGGACTTCACACAGCAC |
| *NR1H3* | GATTTGGACAGTGCCTTGGT | CGCAGAGTCAGGAGGAATGT |
| *NR1H4* | CAGAGCCAAGGAAGAGATGC | CAATGAGGTGAGGAGGAGGA |
| *NR1l2* | GGCATGAAGAAGGAGATGAT | TGGGAGAAGGTAGTGTCAAA |
| *SCARB1* | GGTCCATCTACCCACCCAAC | CAGCGTTGAGGAAGTGAGGAT |
| *SLC10A1* | ATCGTCCTCAAATCCAAACG | TGGCAGAGAGAACTGTGACG |
| *SLC51B* | TGCTGGAAGAGATGCTTTGGT | CTGCTTGCCTGGATGCTTCT |
| *SLCO1B1* | ACTGATTCTCGATGGGTTGG | TATTTGGAGTTTGGGGCA |
| *SQLE* | GCTTCCTTCCTCCTTCATCAGT | AAGCAACAGTCATTCCTCCACC |
| *SREBF1* | CCTGGTCATCTCACAGCAAA | GGCCTTTCACAGAACAGGAA |
| *SREBF2* | AGGCAGGCTTTGAAGACGAA | GTACATCGGAACAGGCGGAT |
| *SULT2A1* | GATCCAATCTGTGCCCATCT | GGGAGGTGGGAGGAGAATAA |
| *UGT1A1* | TGGAATCAACTGCCTTCACCA | TGAGACCATTGATCCCAAAGAGA |
| *UGT2B4* | GAAGTTCTAGGAAGACCCACTACG | GGGTGAGGAAATTGAAAATCCCAG |
| *UGT2B7* | TGACATGAAGAAGTGGGATCAGT | AGCCATACGTCAGCTTTCCC |
| *VLDLR* | TGGACTGATGCGGCTTCTAA | CTATGGAGGCAGGCTCTCG |
| *GAPDH* | ATTTGGCTACAGCAACAGGG | CAACTGTGAGGAGGGGAGA |
